# Efficacy of bergamot: From anti‐inflammatory and anti‐oxidative mechanisms to clinical applications as preventive agent for cardiovascular morbidity, skin diseases, and mood alterations

**DOI:** 10.1002/fsn3.903

**Published:** 2019-01-25

**Authors:** Simone Perna, Daniele Spadaccini, Leonardo Botteri, Carolina Girometta, Antonella Riva, Pietro Allegrini, Giovanna Petrangolini, Vittoria Infantino, Mariangela Rondanelli

**Affiliations:** ^1^ Department of Biology College of Science University of Bahrain Zallaq Bahrain; ^2^ Department of Public Health, Experimental and Forensic Medicine Section of Human Nutrition, Endocrinology and Nutrition Unit Azienda di Servizi alla Persona University of Pavia Pavia Italy; ^3^ Department of Earth and Environmental Sciences Mycology and Plant Pathology Laboratory Pavia Italy; ^4^ Research and Development Unit Indena Milan Italy; ^5^ Department of Biomedical Science and Human Oncology University of Bari Bari Italy; ^6^ IRCCS Mondino Foundation Pavia Italy

**Keywords:** bergamot, bone, cardiovascular, diabetes, inflammation, skin

## Abstract

We summarize the effects of bergamot (extract, juice, essential oil, and polyphenolic fraction) on cardiovascular, bone, inflammatory, skin diseases, mood alteration, anxiety, pain, and stress. This review included a total of 31 studies (20 studies on humans with 1709 subjects and 11 in animals (rats and mice)). In humans, bergamot‐derived extract (BE) exerts positive effects on hyperlipidemia with an oral dose from 150 mg to 1000 mg/day of flavonoids administered from 30 to 180 days, demonstrating an effect on body weight and in modulating total cholesterol, triglycerides, LDL, and HDL. Studies in animals confirm promising data on glucose control (500/1000 mg/day of BE with a treatment lasting 30 days) are available in rats. In animals models, bergamot essential oil (BEO, 10 mg/kg or 20 mg/kg daily for 20 weeks) increases bone volume, decreases psoriatic plaques, increases skin collagen content, and promotes hair growth. Bergamot juice (20 mg/kg) is promising in terms of pro‐inflammatory cytokine reduction. In humans, aromatherapy (from 15 to 30 min) does not appear to be useful in order to reduce stress, anxiety, and nausea, compared to placebo. Compared to baseline, BE topical application and BEO aromatherapy reduce blood diastolic and systolic pressure and could have a significant effect on improving mental conditions.

**Abbreviations**
BE: Bergamot extract
BEO: Bergamot essential oil
BJ: Bergamot juice
BPF: Bergamot polyphenolic fraction
CAF: Cafeteria diet
SC: Standard chow

## 1 INTRODUCTION

Bergamot is the common name of the *Citrus bergamia Risso et Poiteau* plant (Navarra, Mannucci, Delbò, & Calapai, 2015). The fruit has a yellow peel and is the size of an orange. Although native to South‐East Asia, 80% of bergamot is produced in Calabria, southern Italy, where it grows extensively. Fresh juice from bergamot has been studied to evaluate the polyphenolic composition by HPLC‐DAD analysis and total polyphenols content by UV method (Picerno et al., 2011). Bergamot essential oil (BEO) and bergamot juice (BJ) contain up to 93–96% of volatile compounds, such as monoterpenes (25–53% of limonene), as well as discrete quantities of linalool (2–20%) and linalyl acetate (15–40%). BEO also presents a variable percentage (4–7%) of nonvolatile compounds, such as pigments, waxes, coumarins, and psoralens (Mannucci et al., 2017). The main preparations used are bergamot extracts (BE), with high content of flavonoids, such as neoeriocitrin, neohesperidin, naringin (Toth et al., 2015), bergamot polyphenolic fraction (BPF) (Bruno, Pandolfo, Crucitti, Maisano, Zoccali, et al., 2017), bergamot essential oil (BEO) (Watanabe et al., 2015), and aromasticks with bergamot/sandalwood or frankincense/mandarin/lavender (Dyer, Cleary, McNeill, Ragsdale‐Lowe, & Osland, 2016) bergamot/vetivert/geranium (Wiebe, 1998), bergamot/lavender/cedarwood (Graham, Browne, Cox, & Graham, 2003) and bergamot juice (BJ) (Impellizzeri et al., 2015), bergamot/boxthorn extract (Shao, 2003) or bergamot essential oil plus other citrus essential oils plus grapefruit juice (Li, Zhu, Han, & Zhang, 2016) or bergamot flavonoids and other phytoextracts (Babish et al., 2016; Saiyudthong & Marsden, 2011).

Literature suggests that bergamot plays an important role on different areas of interest as nervous system, cardiovascular health, inflammation, diabetes bone metabolism, and skin. Preliminary results show that BEO extract may reduce cardiovascular disease (Lopez, Mathers, Ezzati, Jamison, & Murray, 2006; Nelson, 2013), anxiety, stress, improvement of the cognitive function, and improvement of the sleep (Dyer et al., 2016; Saiyudthong & Marsden, 2011).

Inflammation also seems to benefit from bergamot administration (Impellizzeri et al., 2015, 2016). Finally, bergamot shows positive effects on psoriasis (Valkova, 2007) and on hair growth (Shao, 2003).

The purpose of this review is to summarize the previously published clinical studies in animals and in humans where the efficacy has been evaluated in terms of dosage and timing of administration of bergamot with regard to the nervous system, cardiometabolic markers, diabetes, inflammation, bone, and skin.

### 1.1 Anti‐inflammatory and anti‐oxidative mechanisms of bergamot derivatives

The anti‐inflammatory potential of BJ has never been evaluated after the 2011. In the study of Risitano et al., 2014 has been demonstrated that the flavonoid fraction is able to reduce protein levels of pro‐inflammatory cytokines (Risitano et al., 2014).

The in vitro anti‐inflammatory activity of flavonoid fraction from bergamot juice, suggesting the activation of SIRT1 and demonstrate the inhibitory effects of BJe on LPS‐induced increases in mRNA transcripts and protein levels of pro‐inflammatory cytokines such as IL‐8 gene expression. (Borgatti et al., 2011; Xie, Zhang, & Zhang, 2013). The antioxidant activity of BJe was focused on the cytoprotective ability of BJe against oxidants, such as hydrogen peroxide (H_2_O_2_) and (Fe_2_SO_4_)_3_, that cause oxidative cell damage (Ferlazzo et al., 2015).

Trombetta et al. (2010) evaluated the antioxidant/anti‐inflammatory activity of two alcoholic flavonoid‐rich extracts from bergamot peel on human vessel endothelial cells (HUVECs) exposed to the pleiotropic inflammatory cytokine TNF‐α, a model of vascular oxidative stress, and they showed that both extracts prevented the oxidative stress induced by TNF‐α, modulated the activation of redox‐sensitive transcription factors NF‐κB, thus increasing the cell survival.

### 1.2 Effective mechanisms of bergamot derivatives on clinical diseases

#### 1.2.1 Lipid‐lowering and cardiovascular risk

This lipid‐lowering effect was associated with significant reductions in biomarkers used to detect vascular oxidative damage (such as malondialdehyde, oxyLDL receptor LOX‐1, and protein kinase B (PKB)), suggesting a multi‐action improved potential for bergamot in patients taking statins (Gliozzi et al., 2013). Furthermore, its lipid and glycemic effects may result in a reduction of CV risk. Additionally, bergamot protects against free radical damage in the body, including the vascular endothelium, an important determinant of CV health; yet, bergamot initiates adenosine monophosphate (AMP)‐activated PK (AMPK), a central regulator of energy, and thus is involved in glucose and fatty acid metabolism.

#### 1.2.2 Reducing the sensation of hunger

Bergamot contains polysaccharides and a fibrous‐woody fraction that can be used in food integrators and in dietary products in order to reduce the sensation of hunger (Giannetti, Mariani, Testani, & D'Aiuto, 2010).

#### 1.2.3 Mechanism of naringin in improving the overall insulin sensitivity and glucose tolerance

Few studies have assessed the effects of bergamot on glycemic parameters, and the current positive results are not to be underestimated. Together with the finding that naringin improved overall insulin sensitivity and glucose tolerance (Mandalari et al., 2007).

#### 1.2.4 Mechanism on synaptic transmission

Microdialysis studies demonstrate that, for systemic administration, BEO increases extracellular aspartate, glycine, and taurine in the hippocampus of freely moving rats via a Ca^2+^‐dependent mechanism; in fact, in experiments carried out with a cerebrospinal fluid devoid of Ca^2+^, the latter effect is suppressed suggesting that the phytocomplex interferes with the exocytotic release of amino acid neurotransmitters (Morrone et al., 2007). BEO stimulates the release of endogenous glutamate as well as of [^3^H]D‐aspartate from pre‐loaded synaptosomes.

Intriguingly enough, under these experimental conditions, microdialysis experiments show that BEO does not affect basal amino acid levels in the frontoparietal cortex (penumbra region), whereas it significantly reduces excitatory amino acid, namely glutamate, efflux typically enhanced in this brain region soon after occlusion of the middle cerebral artery (Amantea et al., 2009).

#### 1.2.5 Mechanism on wound healing activities

Bergamot oil and its major active components, namely limonene, linalyl acetate, and linalool, have demonstrated anti‐inflammatory, immunomodulatory, and wound healing activities under different conditions. Cilantro oil and its major active component, linalool, have also been reported to possess anti‐inflammatory and wound healing properties. Of note, a literature search revealed no published studies of spikenard or its major active components in human cells or their anti‐inflammatory and wound healing activities (Han, Beaumont, & Stevens, 2017; Han, Gibson, Eggett, & Parker, 2017).

## 2 MATERIALS AND METHODS

**Figure 1:**
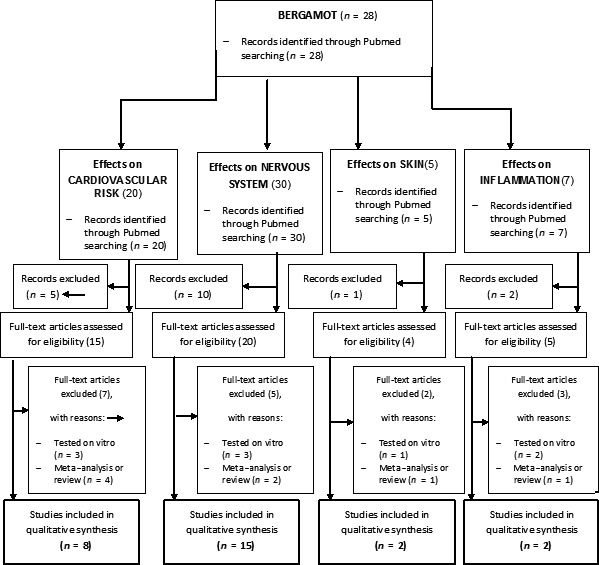
Flow chart of the study

The present systematic review was performed according to the steps by Egger, Davey‐Smith, and Altman (2008) as follows:

1 Configuration of a working group: Three operators were skilled in the effects of bergamot in health, of whom one was acting as a methodological operator and two were participating as clinical operators;
2 Formulation of the revision question on the basis of considerations made in the abstract: “the state of the art on evidence regarding bergamot effects on animals and humans studies”;
3 Identification of relevant studies: A research strategy was planned, on PubMed, taking into account the following varieties:
  (a) definition of the key words (bergamot OR *citrus bergamia* AND skin OR cholesterol OR triglycerides OR inflammation OR glycaemia OR bone mineral density OR stress OR nervous OR anxiety OR osteoporosis OR cardiovascular), allowing the definition of the interest field of the documents to be searched and used separately or in combination;
  (b) use of the Boolean AND operator, which allows the establishment of logical relationships among concepts;
  (c) research modalities: advanced search;
  (d) limits: papers published until 2018 in humans and animals; languages: English;
  (e) manual search performed by the senior researchers, as shown in Table 3;
4 Analysis and presentation of the data: The data extrapolated from the revised animal and human studies were collocated in different tables representing specific areas of interest; in particular, for each study we specified the author and year of publication, study characteristics with main results. The flow diagram of narrative review of the literature has been reported in Figure 1.

## 3 RESULTS: EFFECTS OF BERGAMOT ON CLINICAL APPLICATION: FROM ANIMAL TO HUMAN

This systematic review included a total of 31 studies. In particular, we have considered 20 studies on humans with 1709 subjects. Eleven studies in animals were considered, in particular for both rats and mice. Figure 1 represents the flowchart of the study selection process.

### 3.1 Effects of bergamot on cardiovascular diseases

Concerning the effects of bergamot (BEO, BPF, BE, BJ, aromatherapy), the literature research based on the keywords “bergamot” and [“cardiovascular” or “cholesterol” or “hypertension” or “hdl” or “tryglicerides” or “ldl” or “weight”] retrieved 20 articles.

After the screening process, 15 papers were selected for full‐text revision. After applying the inclusion and exclusion criteria, seven studies were excluded and eight studies both on humans (6) with 448 subjects and in animals (2) were selected for the present systematic review.

#### 3.1.1 Effects on humans

Table 1 shows human studies and the effects of bergamot on cardiovascular markers. In particular, improvements in hypercholesterolemia, triglyceridemia, and weight management are shown.

**Table 1:** Bergamot effects on cardiometabolic markers

Article	Area of interest	Type of study level of evidence	Compound	Sample	Posology and treatment	Main results
In human						
Toth et al. (2015)	Plasma Lipids	Prospective study (level 2)	Bergamot extract: Flavonoids (neoeriocitrin, neohesperidin, naringin)	42 men and 38 women, mean age: 55 ± 13 years	150 mg of flavonoids, with 16% of neoeriocitrin, 47% of neohesperidin, and 37% of naringin) daily for 6 months.	Reduced total cholesterol (*p* < 0.0001), triglycerides (*p* = 0.002), LDL cholesterol (*p* < 0.0001); HDL cholesterol increased (*p* = 0.0007)
Bruno, Pandolfo, Crucitti, Cacciola, et al. (2017), Bruno, Pandolfo, Crucitti, Cedro, et al. (2017), Bruno, Pandolfo, Crucitti, Maisano, et al. (2017)	Metabolic parameters in subjects receiving second generation antipsychotics (SGA)	Open‐label, preliminary study (level 3)	Bergamot polyphenolic fraction (BPF)	28 (15M/13W) mean age 45.8 ± 11.7 outpatient patients treated with SGA	500 mg/day for 60 days	No significant differences in other clinical and metabolic parameters (total, HDL, LDL cholesterol, glucose, systolic and diastolic pressure) were observed (*p* = NS)
Bruno, Pandolfo, Crucitti, Cacciola, et al. (2017), Bruno, Pandolfo, Crucitti, Cedro, et al. (2017), Bruno, Pandolfo, Crucitti, Maisano, et al. (2017)	Metabolic parameters in a sample of subjects receiving second generation antipsychotics (SGA)	Pilot study (level 3)	Bergamot polyphenolic fraction (BPF)	15 outpatients, (9 M/6 W), aged (44.5 ± 9.1), in treatment with SGAs	BPF 1000 mg/day for 30 days	BPF administration resulted in a statistically significant reduction of body weight (*p* = 0.004) and body mass index decrease (*p* = 0.005). No significant differences in other clinical and metabolic parameters were observed
Mollace et al. (2011)	Hypolipidemic and hypoglycemic activity	Randomized, double‐blind, placebo‐controlled (level 1)	Bergamot polyphenolic fraction (BPF)	237 patients with hyperlipemia either associated or not with hyperglycemia	BPF (500 and 1000 mg daily) given orally for 30 days	Bergamot polyphenol (500 mg) fraction (BPF) reduces total cholesterol, LDL cholesterol, and triglycerides and increases HDL, compared to baseline (*p* < 0.0001). Differences between 500 mg and 1000 mg dosage were statistically significant only for HDL (*p *<* *0.05)
Gliozzi et al. (2013)	Hypolipidemic and vasoprotective response in patients with mixed hyperlipidemia	A prospective, open‐label, parallel group, placebo‐controlled study (Level 2)	Bergamot‐derived polyphenolic fraction (BPF)	77 patients with elevated serum LDL‐C and triglycerides	Patients randomly assigned to a group receiving placebo, a group with BPF alone (1000 mg/daily) for 30 days, given orally; and a group receiving BPF (1000 mg/daily given orally) plus rosuvastatin (10 mg/daily for 30 days)	Oral administration of BPF (1000 mg/daily for 30 consecutive days) significantly reduced total cholesterol, LDL‐C, triglyceride and enhanced HDL‐C levels. (*p* < 0.05 vs. placebo)
Babish et al. (2016)	Moderate cardiometabolic risk factors	Pilot observational human study (level 3)	Bergamot Fruit extract (BFE)	11 subjects (3M/8F) with moderate dyslipidemia	2 capsules of Cardiox LDL, which contains 250 mg of BFE (30% bergamot flavonoids), and 110 mg of a blend of nine other phytoextracts (total 500 BFE and 220 mg phytocomplex blend), given daily at dinner time for 12 weeks	Decrease noted in total cholesterol, LDL cholesterol, and apolipoprotein B (*p* = ns). A post hoc analysis of eight subjects with HbA1c > 5.4 and HOMA‐IR score > 2 or elevated triglycerides revealed in addition a decrease in triglycerides, and plasminogen activator inhibitor type 1 (PAI‐1). (*p* < 0.05)

Article	Main effects	Type of study level of evidence	Compound	Sample	Posology	Main results
In vivo						
Mollace et al. (2011)	Hypolipidemic and hypoglycemic activity	Animal (level 6)	Bergamot polyphenol fraction (BPF)	Wistar rats	Administration of BPF (10 or 20 mg/kg/daily for 30 days	Bergamot polyphenol fraction significantly reduces total cholesterol (tChol), LDL cholesterol (cLDL), and triglycerides (TG) and enhances fecal sterols excretion (*p* < 0.05 compared to control rats kept on high‐cholesterol diet
Parafati et al. (2015)	Nonalcoholic fatty liver disease (NAFLD) outcomes	*Animal (level 6)*	(BPF) bergamot polyphenol fraction	RATS	BPF treatment (50 mg/kg/day supplemented with drinking water, 3 months) with two types of diets: Standard chow (SC) and cafeteria diet (CAF)(15% protein, 70% carbohydrates, 15% fat).	BPF, in association with CAF diet, reduces total cholesterol (*p* < 0.05), triglycerides (*p* < 0.01), LDL (*p* < 0.05) and weight gain (*p* < 0.05), compared to CAF diet only. No significant reduction of these parameters was observed in SC + BPF, compared to SC.

Studies suggest a cardio‐protective effect of a single daily dose for 6 months of BE (150 mg of flavonoids, with 16% of neoeriocitrin, 47% of neohesperidin, and 37% of naringin) (Toth et al., 2015) in subjects with hypercholesterolemia with a reduction of total cholesterol (*p* < 0.0001), triglycerides (*p* = 0.002), and LDL cholesterol (<0.0001), while HDL cholesterol increased (*p* = 0.0007).

A recent 2017 study performed by Bruno et al. tested the efficacy and safety of BPF on the improvement of metabolic parameters in subjects receiving second‐generation antipsychotics (SGA). In particular, two subsequent studies by Bruno et al. tested two different doses. In the first study (daily dose of 1000 mg/day for 30 days) (Bruno, Pandolfo, Crucitti, Maisano, et al., 2017), the treatment produced a statistically significant reduction of body weight (*p* = 0.004), in addition to the body mass index decrease (*p *= <0.05). In the second study, (daily dose of 500 mg/day for 60 days) the treatment did not show significant result (Bruno, Pandolfo, Crucitti, Cacciola, Santoro, et al., 2017).

In 2016, (Babish et al. (2016) showed that a 250 mg dose of bergamot fruit extract (BFE) and 110 mg of a blend of nine other phytoextracts showed a decrease in total cholesterol, LDL, and apolipoprotein B (*p* < 0.05). A post hoc analysis showed other significant effects only in eight subjects with HbA1c > 5.4 and HOMA‐IR score >2 or elevated triglycerides: reduction in triglycerides and plasminogen activator inhibitor type 1 (PAI‐1) (*p* < 0.05).

In two recent Italian clinical trials performed for 30 days, BPF at a daily dose of 500 mg or 1000 mg resulted in reducing total cholesterol, LDL, and triglycerides, and increasing HDL, compared to baseline (*p* < 0.0001). Furthermore, it showed a dose‐dependent difference after 500 mg and 1000 mg only for HDL (*p* < 0.05) (Mollace et al., 2011).

In a similar study by Gliozzi, an oral administration of 1000 mg daily of BPF significantly reduced total cholesterol, LDL, and triglycerides, while enhancing HDL levels, compared to placebo group (*p* < 0.05) (Gliozzi et al., 2013).

#### 3.1.2 Effects on animal models

In literature, there are poor results regarding the effect of bergamot on cardiovascular diseases in animals. A study was conducted on rats using BPF (10 or 20 mg/kg/daily for 30 days), reporting a reduction in total cholesterol, LDL, and triglycerides, enhancing fecal sterols excretion, compared to controls (*p* < 0.05) (Bruno, Pandolfo, Crucitti, Cedro, Zoccali, et al., 2017).

Even an administration of 50 mg/kg/daily of BPF for 30 days in combination with a hyperlipidic and hyperglycemic diet (cafeteria diet, CAF) reduced significantly total cholesterol (*p* < 0.05), triglycerides (*p* < 0.01), LDL (*p* < 0.05), and weight gain (*p* < 0.05) in rats, in comparison with CAF diet only. In addition, the same administration in association with a standard chow (SC) regime did not produce the same effect (*p *= ns) (Parafati et al., 2015).

### 3.2 Effects of bergamot on diabetes

Concerning the effects of BE, BPF, or BJ, the literature search was based on the keywords “bergamot” and [“DIABETES” OR “GLUCOSE” OR “GLYCEMIA”] and the examination retrieved five articles.

After screening, four papers were selected for full‐text revision. After applying the inclusion and exclusion criteria, two studies were excluded and two studies were selected for the present systematic review.

In Table 2, we have included studies on the effects of bergamot on diabetes for a total of two studies divided, respectively, into (1) on humans (237 subjects) and (1) on animals.

**Table 2:** Bergamot effects on diabetes

Article	Main effects	Type of study level of evidence	Compound	Sample	Posology	Main results
Human						
Mollace et al. (2011)	Hypolipidemic and hypoglycemic activity	Randomized, double‐blind, placebo‐controlled (level 1)	Bergamot polyphenolic fraction (BPF)	237 patients with hyperlipemia either associated or not with hyperglycemia	BPF (500 and 1000 mg daily) given orally for 30 days	Bergamot polyphenol (500 mg) fraction (BPF) reduces blood glucose (Bglucose) levels compared to baseline. (*p* < 0.0001)
Vivo						
Parafati et al. (2015)	Nonalcoholic fatty liver disease (NAFLD) outcomes	Animal (level 6)	(BPF) bergamot polyphenol fraction	RATS	BPF treatment (50 mg/kg/day supplemented with drinking water, 3 months) with 2 types of diets: Standard chow (SC) and cafeteria diet (CAF) (15% protein, 70% carbohydrates, 15% fat)	BPF has a moderate effect on blood glucose. It is shown that BPF reduces significantly blood glucose levels only in association with CAF diet, compared to CAF diet only (*p* < 0.05). While it has no effect in association with SC (*p *= ns)

#### 3.2.1 Effects on humans

The study by (Mollace et al. (2011) on 237 subjects was focused on blood glucose levels. After a daily supplementation of 500 mg for 30 days, BPF resulted in a significant reduction on blood glucose levels compared to baseline (*p* < 0.0001).

#### 3.2.2 Effects on animals

(Parafati et al. (2015) on rats showed that BPF reduces significantly blood glucose levels only in association with CAF diet, compared to CAF diet only (*p* < 0.05), while it has no effect in association with SC diet, compared to SC alone (*p *= ns).

### 3.3 Effects of bergamot on nervous system

Concerning the effects of bergamot, the literature search was based on the keywords “bergamot” and [“NERVOUS SYSTEM” OR “STRESS” OR “ANXIETY”]. The search retrieved 30 articles.

After screening, 20 papers were selected for full‐text revision. After applying the inclusion and exclusion criteria, four studies were excluded and 16 studies were selected for the present systematic review.

Table 3 includes studies on the effects of bergamot on the nervous system, for a total of 16 studies divided, respectively, into (11) on humans (831 subjects) and (5) on animals.

**Table 3:** Bergamot effects on nervous system

Paper	Area of interest	Type of study level of evidence	Compound	Sample	Posology	Main results
In human						
Watanabe et al. (2015)	Psychological stress and anxiety	Random crossover study design (level 2)	Bergamot Essential Oil (BEO)	41 Women	Volunteers exposed to three experimental setups (rest (R), rest + water vapor (RW), rest + water vapor + bergamot essential oil (RWB)) for 15 min each (one‐time intervention)	Salivary cortisol (CS) of all three conditions R, RW, and RWB were found to be significantly distinct (*p* = 0.003). CS value of RWB was significantly lower when compared to the R setup (*p* = 0.004) but also CS in RW group was significantly lower compared to R (*p* = 0.049); CS value in RWB group was not significantly lower than RW (*p *= NS)
Han, Beaumont, et al. (2017), Han, Gibson, et al. (2017)	Mental health and well‐being	A Pilot Study (level 3)	Bergamot Essential Oil (BEO)	57 participants (50 W/7 M, age range: 23–70 years)	15 min of bergamot essential oil exposure or distilled water aromatherapy (one‐time intervention)	PANAS survey showed near significant differences between groups in proudness (*p* = 0.07), activeness (*p* = 0.07) but also nervousness (*p* = 0.06)
Ni et al. (2013)	Preoperative anxiety	A randomized controlled trial (level 1)	Bergamot Essential Oil (BEO)	109 (mean age 45.4 ± 11.7) preoperative patients (44 M/65 F)	30 min of bergamot essential oil aromatherapy or water vapor (one‐time intervention)	State Trait Anxiety Inventory (STAI) was significantly different between groups both in patients without a previous experience of surgery (*p* = 0.021) and patient with a previous experience of surgery (*p* = 0.005)
Bruno, Pandolfo, Crucitti, Cacciola, et al. (2017), Bruno, Pandolfo, Crucitti, Cedro, et al. (2017), Bruno, Pandolfo, Crucitti, Maisano, et al. (2017)	Cognitive/executive functioning	Open‐label pilot (level 3)	Bergamot polyphenolic fraction (BPF)	20 outpatients, 15 (M) and 5 (W). Aged 20–58 years	BPF at an oral daily dose of 1000 mg/d for 8 weeks	BPF supplementation significantly improved Wisconsin Card Sorting Test (WCST) “perseverative errors” (*p* = 0.004) and semantic fluency test (*p* = 0.004)
Liu et al. (2013)	Work‐related stress	Nonrandomized controlled trial (level 2)	Bergamot essential oil (BEO)	29 Taiwanese elementary school teachers (3M/26F), mean age 41.4 ± 4.	15 min of bergamot essential oil diluted to 2% aromatherapy (one‐time intervention)	Aromatherapy treatment with bergamot reduces low‐frequency power (LF) (*p* < 0.001), LF/HF ratio (*p* < 0.001), and LF% (*p* < 0.001) and increases high‐frequency power % (*p* < 0.001)
Dyer et al. (2016)	Improving sleep	Prospective Study (level 2)	Bergamot (*Citrus bergamia*) aromasticks	65 patients, 11 (M) mean age (52),54 (F) mean age (84)	Use the aromastick for a minimum of two nights.	64% of patients showed a improvement of at least one point in sleep following the use of an aromastick
Hongratanaworakit (2011)	Autonomic parameters and emotional responses in humans following transdermal absorption	No randomized trial (level 2)	Essential oil: bergamot and lavender	40 healthy volunteers, 17 (M), 23 (F). Mean age (24.95 ± 6.67)	1 ml of a 10%, w/w, solution of blended essential oil was applied topically to the skin of the lower abdomen of each subject with a sel‐massage of 5 min (one‐time intervention)	Was observed a significant decrease of systolic blood pressure (SBP) (*p* = 0.014), diastolic blood pressure (DBP) (*p* = 0.007) and pulse rate (PR) *p *= (0.033) in the blended essential oil group compared to placebo group. The subjects of essential oil group compared to the placebo group appeared more relaxed based on a verbal scale (*p* = 0.021)
Ndao et al. (2012)	Anxiety, nausea, and pain in pediatric patients poststem cell transplantation (SCT)	Double‐blind, placebo‐controlled randomized study (level 1)	Bergamot essential oil (BEO)	37 pediatric patients, 10 (F), 27 (M), mean age (13.8 ± 4.9)	Four drops of diffused essential oil per hour (duration: 3 h) The placebo was a non‐essential oil‐based scented shampoo.(one‐time intervention)	Spielberger State‐Trait Anxiety Inventory (STAI) was used to report anxiety, while Visual analog scale (VAS) was used to report Nausea. Patients in treatment group experienced more anxiety than control group after treatment (t3: *p* = 0.01; t4: *p* = 0.05) Also nausea remained greater among treatment group. (t3: *p* < 0.01; t4: *p* = 0.03)
Chang and Shen (2011)	Autonomic nervous system regulation	Nonrandomized trial (level 2)	Bergamot essential oil (BEO)	54 elementary school teachers (25M/29F) aged >35	Bergamot essential oil 2% for aromatherapy spray for 10 min (one‐time intervention)	Aromatherapy significantly decreases blood systolic and diastolic pressure (*p* < 0.001), heart rate (*p* < 0.001), low‐frequency power (LF) (*p* < 0.01), and LF/HF ratio (*p* < 0.01); increases heart rate variability (HRV) (*p* < 0.001) and high‐frequency power (HF) (*p* < 0.01)
Wiebe (1998)	Preoperative anxiety	A double‐blind, randomized trial (level 1)	Essential oils: vetivert, bergamot, and geranium	66 women	10 min of aromatherapy: essential oils vetivert, bergamot, and geranium (treatment) or a hair conditioner (placebo) (one‐time intervention)	Anxiety measured with a verbal scale was reduced after exposure both in treatment and placebo group, with no significant differences between groups (*p *= ns)
Graham et al. (2003)	Anxiety during radiotherapy	Placebo‐controlled double‐blind randomized trial (level 1)	Carrier oil with fractionated oils, carrier oil only, or pure essential oils of lavender, bergamot, and cedarwood	313 patients (163 M/150W)	Three drops of oil were applied to the bib, the duration of exposure was 15–20 min (one‐time intervention)	There were no significant differences in HADS (hospital anxiety and depression scale) or SPHERE (somatic and psychological health report) scores between the randomly assigned groups. However, HADS anxiety scores were significantly lower at treatment completion in the non‐fragrant placebo group (carrier oil only), compared with either of the fragrant arms (*p* = 0.04).
In vivo						
Saiyudthong and Marsden (2011)	Anxiety‐related behaviors and stress‐induced levels of plasma corticosterone	Animal (level 6)	Bergamot essential oil (BEO) and diazepam	In rats	Inhalation of BEO (1%/2.5%/5%) for 7 min and injection of diazepam (1 mg/kg, i.p.) (one‐time intervention)	Both BEO 2.5% and diazepam exhibited anxiolytic‐like behaviors (open arms time, *p* < 0.01 and entries, *p* < 0.05) and reduced plasma corticosterone (*p* < 0.05). BEO 1% and 5% did not reduce plasma corticosterone. (*p *= ns)
Amantea et al. (2009)	Prevention in glutamate accumulation and neuroprotection	Animal (level 6)	Bergamot essential oil (BEO; Citrus bergamia, Risso)	In rats	Bergamot essential oil (BEO) 0.1–0.5 ml/kg given intraperitoneally 1 h before induced ischemia (one‐time intervention)	Microdialysis experiments show that BEO (0.5 ml/kg) did not affect basal amino acid levels (*p* = ns) but significantly reduced excitatory amino acid efflux in the frontoparietal cortex (*p* < 0.05)
Lauro et al. (2016)	Inhibition of morphine tolerance	Animal (level 6)	Bergamot polyphenolic fraction (BPF)	Male mice	Injection of different doses of BPF (5, 25, and 50 mg/kg) twice a day, followed by a subcutaneous injection of morphine (20 mg/kg) for 5 days. At fifth day, morphine injection was 3 mg/kg.	Co‐administration of morphine with BPF (5–50 mg/kg) inhibited the development of morphine tolerance in a dose‐dependent manner (*p* < 0.01 for 5 mg/kg and *p* < 0.001 for other doses, compared to morphine alone)
Rombolà et al. (2017)	Anxiolytic/sedative‐like effects	Animal (level 6)	Bergamot essential oil (BEO), benzodiazepine diazepam (DZP) jojoba oil	Male rats	Intraperitoneal injections of BEO (100, 250, or 500 μl/kg) DZP (1.2 or 5 mg/kg) or jojoba oil (500 μl/kg). For the lowest doses of bergamot oil, the total volume injected was 500 μl/kg by adding jojoba oil, an unscented oil used as vehicle. (one‐time intervention)	BEO 250 μl/kg and 500 μl/kg, compared to jojoba oil, respectively, decreases grooming in rats (*p* < 0.001/*p* < 0.0001) and increases immobility (*p* < 0.05/*p* < 0.001). A statistically significant difference is also observed at 5 min between BEO 500 μL/kg and DZP 1.2 mg/kg (*p* < 0.05)
Rombolà et al. (2009)	Gross behavior and EEG spectrum	Animal (level 6)	Bergamot essential oil (BEO)	Male rats	Systemic administration of BEO 100, 250, 500 μl/kg (one‐time intervention)	Systemic administration of increasing volumes of BEO was found to induce dose‐dependent active behavior and EEG background activity in the hippocampus and in the cortex.

#### 3.3.1 Effects on humans

Literature shows several studies in humans regarding the inhalation of bergamot with controversial results on the nervous system and mood.

In particular, 15 min of BEO vapor inhalation by healthy females was tested in comparison with water vapor inhalation or rest (control). Promising results showed that salivary cortisol (CS) after inhalation was significantly different in the three groups (*p* = 0.003) and CS levels were lower compared to control group in both BEO vapor and water vapor group (*p* = 0.004 and *p* = 0.0049), but CS in BEO vapor group was not significantly lower than in respect of CS in water vapor group (Watanabe et al., 2015).

Similarly, (Ni et al. (2013) tested BEO aromatherapy on preoperative patients to evaluate effects on preoperative anxiety. In this case, the State Trait Anxiety Inventory (STAI) scoring after 30 min of inhalation was significantly different between BEO and the water vapor group, both in patients without previous experience of surgery (*p* = 0.021) and patients with previous experience of surgery (*p* = 0.005).

BEO was also tested in comparison with other fragrant non‐essential oil aromatherapies. In the study by (Ndao et al. (2012)), in pediatric patients poststem cell transplantation (SCT), the BEO aromatherapy experienced more anxiety (t3: *p* = 0.01; t4: *p* = 0.05) and nausea (t3: *p* < 0.01; t4: *p* = 0.03) after inhalation, compared to control group.

Two other randomized controlled trials showed similar results. (Wiebe (1998) tested preoperative anxiety in six women with a 10 min aromatherapy based on a mixture of essential oils (vetivert, bergamot, and geranium) in comparison with a placebo group, which inhaled a hair conditioner. In this study, anxiety was reduced after exposure to both treatment and placebo, with no significant differences between groups.

(Graham et al. (2003) showed that in patients subjected to radiotherapy and the effects of essential oils (lavender, bergamot, and cedarwood), there were no significant differences in HADS (hospital anxiety and depression scale) or SPHERE (somatic and psychological health report) scores between the randomly assigned groups. However, HADS scores were significantly lower at treatment completion in the non‐fragrant placebo group (carrier oil only), compared with either of the fragrant arms (*p* = 0.04).

Effects of bergamot on the nervous system were also evaluated in terms of blood pressure, heart variability, and pulse rate. In particular, (Liu, Lin, & Chang (2013) evaluated if BEO aromatherapy (2% diluted, 15 min), in Taiwanese teachers, could ease work‐related stress. Compared to baseline values, BEO reduced low‐frequency power (LF), LF%, LF/HF ratio (*p* < 0.001) and increased high‐frequency power % (HF%) (*p* < 0.001).

Another study by Chang & Shen (2011) showed similar results in elementary school teachers. BEO aromatherapy (2% diluted, 10 min) significantly modulated the autonomic nervous system by decreasing systolic and diastolic pressure (*p* < 0.001), heart rate (*p* < 0.001), LF (*p* < 0.01), LF/HF ratio (*p* < 0.01) and increasing heart rate variability (HRV)(*p* < 0.001) and HF (*p* < 0.01). Finally, the randomized placebo‐controlled study by Hongratanaworakit (2011) investigated 40 healthy volunteers through the application of BEO and lavender essential oil on skin. After the topical application of the oil, 1 ml (10% diluted), on the lower abdomen of subjects, a significant decrease in systolic blood pressure (SBP) (*p* = 0.014), diastolic blood pressure (DBP) (*p* = 0.007), and pulse rate (PR) *p *= (0.033) was observed in the blended essential oil group compared to placebo group. The subjects of the essential oil group compared to the placebo group appeared more relaxed based on a verbal scale (*p* = 0.021).

A recent study by Han, Gibson, et al. (2017) evaluated the effects of bergamot on mental health through the use of approved tests: BEO aromatherapy effects on mental health and well‐being in comparison with distilled water aromatherapy (15 min) in 57 subjects. Positive and Negative Affect Schedule (PANAS) survey showed near significant differences between groups in proudness (*p* = 0.07), activeness (*p* = 0.07) but also nervousness (*p* = 0.07).

Another study by Bruno, Pandolfo, Crucitti, Cedro, et al. (2017) showed significant effects in cognitive/executive functioning in psychiatric patients with an oral dose of BPF (1000 mg/day for 8 weeks) that significantly improved Wisconsin Card Sorting Test (WCST) “perseverative errors” (*p* = 0.004) and semantic fluency test (*p* = 0.004).

Bergamot was also tested in terms of sleep improving using bergamot/sandalwood or frankincense/mandarin/lavender essential oil aromasticks for a minimum of two nights in rooms. About 64% of patients showed an improvement of at least one point in sleep following the use of an aromastick. (*p *= NR) (Dyer et al., 2016).

#### 3.3.2 Effects on animals

Several studies investigated the effects of bergamot on mental health and nervous system in vivo using BEO (diluted 1%, 2.5% and 5%) and showed different results. BEO 2.5% and diazepam (1 mg/kg) both exhibited anxiolytic‐like behaviors in rats (open arms time, *p* < 0.01 and entries, *p* < 0.05) and reduced plasma corticosterone (*p* < 0.05). BEO 1% and 5% did not reduce plasma corticosterone (Saiyudthong & Marsden, 2011). Rombolá et al. (2009) studied modifications in male rats’ gross behavior and EEG spectrum with a systemic administration of increasing volumes of BEO (100, 250, 500 μl/kg). It was found that this administration schedule induced dose‐dependent active behavior and EEG background activity in the hippocampus and in the cortex (*p *= ns).

Intraperitoneal injections of BEO (250 and 500 μl/kg), compared to jojoba oil (500 μl/kg) (Rombolà et al., 2017), respectively, decreased grooming in rats (*p* < 0.001/*p* < 0.0001) and increased immobility (*p* < 0.05/*p* < 0.001).

Amantea et al. (2009) tested BEO (0.1 and 0.5 ml/kg) to prevent glutamate accumulation and neuroprotection in microdialysis experiments. BEO 0.5 ml/kg did not affect basal amino acid levels but significantly reduced excitatory amino acid efflux in frontoparietal cortex (*p* < 0.05). Injections of different doses of BPF (5, 25, and 50 mg/kg) also inhibited the development of morphine tolerance in a dose‐dependent manner (*p* < 0.01 for 5 mg/kg and *p* < 0.001 for other doses, compared to morphine alone) (Lauro et al., 2016).

### 3.4 Effects of bergamot on bone

Concerning the effects of bergamot extracts on bone, the literature search was based on the keywords “bergamot” and [“BONE” or “osteoporosis” or “osteoclasts” or “osteoblasts” or “osteopenia”].

Literature retrieved two articles. After screening, one paper was selected for full‐text revision. After applying the inclusion and exclusion criteria, only one study was selected for the present systematic review. Table 4 shows the study description with main results.

**Table 4:** Bergamot bone effects

Paper	Main effects	Type of study level of evidence	Compound	Model	Posology	Main results
Vivo						
Li et al. (2016)	Improve of diabetes‐related osteoporosis	Animal (level 6)	Bergapten (bergamot essential oil + other citrus essential oils +grapefruit juice)	C57/B6 mice (*n* = 102)	Bergapten orally administered 10 mg/kg or 20 mg/kg daily for 20 weeks	Bergapten increases Bone volume/trabecular volume ratio (BV/TV, trabecular thickness and trabecular number) (*p* < 0.01 for 10 mg/kg and *p* < 0.001 for 20 mg/kg)

#### 3.4.1 Effects in animals

The oral administration of Bergapten (BEO) at 10 or 20 mg/kg daily for 20 weeks was able to significantly increase bone volume/trabecular volume ratio (BV/TV), trabecular thickness, and trabecular number (*p* < 0.01 for 10 mg/kg and *p* < 0.001 for 20 mg/kg) (Li et al., 2016).

### 3.5 Effects of bergamot on inflammation

Concerning the effects of bergamot extract on inflammation, the literature search was based on the keywords “bergamot” and [“inflammation” or “ESR” or “CRP” or “citokine”].

Literature retrieved five articles. After screening, four papers were selected for full‐text revision. After applying our inclusion and exclusion criteria, two studies were excluded and two studies (in vivo) were selected for the present systematic review. Table 5 shows results of the studies selected process.

**Table 5:** Bergamot effects on inflammation

Paper	Main effects	Type of study level of evidence	Compound	Model	Posology	Main results
Vivo						
Impellizzeri et al. (2015)	The effects of BJE in mice subjected to experimental colitis	Animal (level 6)	Bergamot juice (BJe)	Mice subjected to experimental colitis	BJe was administered daily orally (at 5, 10 and 20 mg/kg) for 4 days	Bje 20 mg/kg reduced in a dose‐dependent colon levels of TNF‐α and IL‐1β in DNBS injected mice (*p* < 0.01). Furthermore, BJe 20 mg/kg reduced the degree of positive staining for nitrotyrosine (*p* < 0.01), levels of NK‐kB p65 (*p* < 0.01) and *p*‐JNK expression (*p* < 0.01)
Impellizzeri et al. (2016)	Modulation of ileum inflammation caused by intestinal ischemia/reperfusion (I/R)	Animal (level 6)	Flavonoid‐rich fraction of bergamot juice (BJe)	Adult CD1 male mice	BJe administered intraperitoneally 20 mg/kg (one‐time intervention)	BJe treatment decreases Myeloperoxidase (MPO) activity, TNF‐α and IL‐1β levels compared to placebo in I/R mice (*p* < 0.05)

#### 3.5.1 Effects in animals

Animals studies show that a daily oral administration of BJ extract (20 mg/kg) in mice subjected to experimental colitis reduced colon levels of TNF‐α, IL‐1ß (*p* < 0.01), NK‐kb p65 (*p* < 0.01), *p*‐JNK (*p* < 0.01), and the degree of positive staining for nitrotyrosine (Impellizzeri et al., 2015).

Similar results were found when studying a BJ extract intraperitoneal administered (20 mg/kg), which reduced myeloperoxidase (MPO) activity, TNF‐α, and IL‐1β (*p* < 0.05) levels compared to placebo in intestinal ischemia/reperfusion (I/R) mice (Impellizzeri et al., 2016).

### 3.6 Effects of bergamot on skin

Concerning the effects of bergamot extract, the literature search was based on the keywords “bergamot” and [“PSORIASIS” OR ““OR “UVB” OR “SKIN”].

Literature review retrieved five articles. After screening, four papers were selected for full‐text revision. After applying the inclusion and exclusion criteria, two studies were excluded and two studies were selected for the present systematic review.

In Table 6, studies on the effects of bergamot on skin were included, for a total of two studies divided, respectively, into 1 on humans (193 subjects) and 1 in vivo.

**Table 6:** Bergamot effects on skin

Paper	Main effects	Type of study level of evidence	Compound	Model	Posology	Main results
In human						
Valkova (2007)	Treatment of psoriasis	Randomized controlled trial (level 1)	Bergamot oil	193 patients (119M/74W) mean age 40.9 ± 0.9	UVB+ bergamot oil applied on the psoriatic plaques 30 min before the procedures. Sessions held three times weekly. (duration : from 5 sessions if no significant improvement, up to 17 sessions)	Treatment with UVB + oil significantly reduces Psoriasis Area and Severity Index (PASI) compared to baseline (*p* < 0.001) and number of procedures compared to UVB treatment only (*p* < 0.05)
In vivo						
Shao (2003)	Effects on skin and hair growth in mice.	Animal (level 6)	Bergamot and boxthorn extract	In rats	The skin on the back of mice was shaved topically and smeared with bergamot and boxthorn extract for 42 days	Compared with control group, the extract from bergamot and boxthorn increases the activity of superoxide dismutase (*p* < 0.05), the content of collagen (*p* < 0.001) of skin and the growth of hair (*p* < 0.001). It decreases the content of malondialdehyde (*p* < 0.05) in the skin of mice

#### 3.6.1 Effects in humans

The clinical study on subjects with psoriasis that received a treatment with UVB + BEO applied on the psoriatic plaques 30 min before the procedures, three times weekly, showed a significant reduction of Psoriasis Area and Severity Index (PASI) compared to baseline (*p* < 0.001) and a reduction in the number of procedures compared to UVB treatment only (*p* < 0.05) (Valkova, 2007).

#### 3.6.2 Effects in animals

As regards animal studies, the topical application of bergamot extract for 42 days increased the activity of superoxide dismutase (*p* < 0.05) and the collagen content (*p* < 0.001), and decreased the content of malondialdehyde (*p* < 0.05) in the skin of mice. It also promoted hair growth significantly (*p* < 0.001) (Shao, 2003).

## 4 DISCUSSION

This systematic review was performed in order to elucidate the main pharmacodynamic activities of bergamot both in animals and in humans. We know that bergamot contains several bioactive, such as monoterpenes, linalool, linalyl acetate, and nonvolatile compounds, or pigments, waxes, coumarins, and psoralen, but the real efficacy in humans and animals represents an important key point for developing future new target remedies in different areas.

The main areas of interest in which literature suggests bergamot's beneficial effects are the nervous system, cardiovascular health, inflammation, diabetes, bone, metabolism, and skin.

This review focuses attention on the pharmacodynamic aspects of bergamot in dosage and timing of supplementation in the form of juice, polyphenolic fraction, extract, or oil (intraperitoneal administration, systemic administration, or aromatherapy).

In particular: as regards the effects on hyperlipidemia, this review summarizes that:

Treatment should involve at least an oral dose of 150 mg/day of flavonoids (Bergamot‐derived extract) for 6 months (Toth et al., 2015) or an oral dose of bergamot polyphenolic fraction (BPF) from 500 to 1000 mg/day for 30/60 days (Bruno, Pandolfo, Crucitti, Cacciola, et al., 2017; Gliozzi et al., 2013; Mollace et al., 2011; Toth et al., 2015) for a reduction of body weight or decrease in total cholesterol, triglycerides, LDL and an increase of HDL. Studies in animals confirm these encouraging results, but only in animals kept on a high fat diet.

The effects of bergamot on cardiovascular outcomes were assessed in eight studies (four with level of evidence 3; one with level of evidence 1; one with level of evidence 2, and two with level of evidence 6) with a total of 448 subjects enrolled. This resume suggests that there is not enough one study of level 1 to state that there is wide scientific evidence of effectiveness of bergamot on reduction of total cholesterol, LDL cholesterol, and triglycerides.

As regards the effects on diabetes, this review summarizes that:

Only one study in literature found a significant effect of 500/1000 mg/day of BPF on reducing blood glucose levels in humans, with treatment lasting 30 days (Mollace et al., 2011). Promising data on glucose control are also available in rats with oral administration of BPF with a treatment of 50 mg/kg/day, but only in concomitance with a high fat diet (Parafati et al., 2015).

This resume suggests that there are not enough two studies of level 1 to state that there is wide scientific evidence of effectiveness of bergamot on reduction of glycaemia.

As regards the effects on nervous system, this review summarizes that:

Effects of bergamot on the nervous system are controversial. Aromatherapy (from 15 to 30 min), involving BEO vapor inhalation, appears to be significantly useful in order to reduce stress (salivary cortisol) compared to rest only (Watanabe et al., 2015) or to reduce anxiety compared to water vapor inhalation (Ni et al., 2013). Other studies report that a 10 min aromatherapy based on bergamot and other essential oils does not significantly reduce anxiety compared to a placebo (hair conditioner) inhalation (Wiebe, 1998). Furthermore, one study in literature shows that BEO vapor aromatherapy appears to be less effective in reducing anxiety and nausea than a scented non‐essential oil shampoo aromatherapy (Ndao et al., 2012). In addition, another study concluded that a 20‐min inhalation of bergamot/lavender/cedarwood essential oil is less effective in reducing anxiety, compared to a non‐fragrant carrier oil inhalation. (Graham et al., 2003) Accordingly to these evidences, we can affirm that BEO aromatherapy, compared to placebo group, does not appear to be useful in order to improve any aspect of mental health.

On the other hand, 10–15 min of BEO aromatherapy shows effects like reducing low‐frequency power, increasing high‐frequency power, and increasing heart rate variability, which are markers of a prevalence of parasympathetic activity (Chang & Shen, 2011; Liu et al., 2013). It was also observed that BEO application on skin reduces blood diastolic and systolic pressure and heart rate (Hongratanaworakit, 2011). The power of these studies is low because they compare results to baseline and not to a placebo group. Studies in animals suggest potential effects on reducing plasma corticosterone (Saiyudthong & Marsden, 2011) and excitatory amino acid efflux in frontoparietal cortex (Amantea et al., 2009).

The effects of bergamot on nervous system were assessed in 16 studies (three with level of evidence 3; five with level of evidence 1; three with level of evidence 2, and five with level of evidence 6) with a total of 831 subjects. There are five studies with level of evidence 1 that underline that there is a good scientific evidence regarding effects of bergamot on nervous system. In particular, studies with level of evidence 1 agree that bergamot essential oil aromatherapy has no effect on mental health compared to other fragrant oils and, in certain situations, appears to be less effective than non‐fragrant oils in reducing anxiety and nausea.

As regards the effects on bone, this review summarizes that:

In animals models, the oral administration of BEO (10 mg/kg or 20 mg/kg daily for 20 weeks) plus other compounds increases bone volume/trabecular volume ratio (BV/TV), trabecular thickness, and trabecular number. These data must be verified in human studies (Li et al., 2016).

This resume suggests that there is not enough one study of level 6 to state that there is wide scientific evidence of effectiveness of bergamot on increase in bone mineral density.

As regards the effects on inflammation, this review summarizes that:

BJ extract (20 mg/kg) for 30 days was tested by both oral and intraperitoneal administration in animals. The results are promising in terms of pro‐inflammatory cytokine reduction and may represent an aid for the treatment of inflammatory bowel disease, but currently no studies in humans have been made (Impellizzeri et al., 2015, 2016). This resume suggests that there is not enough one study of level 6 to state that there is wide scientific evidence of effectiveness of bergamot on humans.

As regards the effects on skin, this review summarizes that:

The BEO application combined with UVB therapy on psoriatic plaques (30 min before the procedures three times weekly) reduces Psoriasis Area and Severity Index. In animals, the topical application of bergamot extract for 42 days increased skin collagen content and promoted hair growth significantly.

It seems that a topical application of Bergamot oil/extract in a certain dosage could play an active role in the skin and in promoting hair growth (Shao, 2003; Valkova, 2007). Further studies in humans are needed to confirm this effectiveness and to set a dosage.

The real effects of bergamot in humans must be better elucidated with further large clinical trials.

The key points that must be addressed for better comprehension of bergamot potentiality are the timing of supplementation, the formulation and source of the compound (juice, extract, oil, or aromatherapy etc.), and the fitting area of efficacy.

The effects of bergamot on skin were assessed in two studies (one with level of evidence 1 and one with level of evidence 6) with a total of 193 subjects. At the current state, there is a poor scientific evidence of effectiveness of bergamot on increases in the activity of superoxide dismutase and the content of collagen of skin.

## CONFLICT OF INTEREST

The authors declare that they do not have any conflict of interest.

## DATA SHARING AND DATA ACCESSIBILITY

All considered studies are available on PubMed and/or Google Scholar database. Text with fees was made available free from University of Pavia online library.

## ETHICAL STATEMENT

This study does not involve any human or animal testing.
